# Early breastfeeding practices contribute to exclusive breastfeeding in Bangladesh, Vietnam and Ethiopia

**DOI:** 10.1111/mcn.13012

**Published:** 2020-04-22

**Authors:** Phuong Hong Nguyen, Sunny S. Kim, Lan Mai Tran, Purnima Menon, Edward A. Frongillo

**Affiliations:** ^1^ Poverty, Health and Nutrition Division International Food Policy Research Institute (IFPRI) Washington, DC USA; ^2^ FHI Solution Hanoi Vietnam; ^3^ Health Promotion, Education, and Behavior University of South Carolina Columbia South Carolina USA

**Keywords:** Bangladesh, cluster randomized trial, early initiation of breastfeeding, Ethiopia, exclusive breastfeeding, Vietnam

## Abstract

Limited evidence exists on the complex relationship among interventions, early initiation of breastfeeding (EIBF), prelacteal feeding and exclusive breastfeeding (EBF). We examined whether early breastfeeding practices are associated with EBF and how much improving EIBF and non‐prelacteal feeding contributes to increased prevalence of EBF. Survey data were collected in 2010 and 2014 as part of impact evaluations of Alive & Thrive (A&T) interventions to improve infant and young child feeding (IYCF) practices in Bangladesh, Vietnam and Ethiopia. Multivariable logistic regression analyses were used to examine effects of interventions and early breastfeeding practices on EBF. Structural equation modelling quantified the direct and indirect effects of interventions (via improving EIBF and non‐prelacteal feeding) on EBF. Although breastfeeding is nearly universal in all three countries (≥98%), delayed initiation of breastfeeding is prevalent (>60%) and prelacteal feeding is common. EIBF alone was not associated with EBF, whereas non‐prelacteal feeding was associated with 1.6–3.5 higher odds of EBF. Intervention exposure affected breastfeeding practices in all three countries; these impacts were amplified among those who practiced EIBF or non‐prelacteal feeding [odds ratio (OR) = 11 and 27.5 in Bangladesh and 6.5 and 11.5 in Vietnam, respectively]. The paths through EIBF and non‐prelacteal feeding explained 13%–18% of the effect of the interventions on EBF. Early breastfeeding practices influence EBF, but interventions aimed only at the initiation and early days of breastfeeding will be inadequate to promote EBF. Social and behaviour change interventions should simultaneously target EIBF, non‐prelacteal feeding and EBF to support optimal breastfeeding practices.

**Key messages**

- Data from three impact evaluations of large‐scale social and behaviour change communication interventions in Bangladesh, Vietnam, and Ethiopia were used to examine relationships among early initiation of breastfeeding (EIBF), prelateal feeds, and exclusive breastfeeding (EBF) and how the interventions affected these relationships.
- Non‐prelacteal feeding was associated with 1.6–3.5 higher odds of EBF. The interventions improved breastfeeding practices, with large amplified effects on EBF among those who practiced EIBF or non‐prelacteal feeding. The pathway from EIBF to non‐prelacteal feeding to EBF explained 13–18% of the intervention effect on EBF.
- Intervention strategies should simultaneously address early breastfeeding practices and sustained exclusive breastfeeding.

## 1 INTRODUCTION

Breastfeeding is essential for growth and development of infants and is one of the most cost‐effective interventions to prevent maternal and child morbidity and mortality (Carroll, Buccini, & Perez‐Escamilla, 2018; Rollins et al., 2016; Victora et al., 2016). Recent work has estimated that 823 000 child deaths and 20 000 maternal deaths could be prevented annually by scaling up breastfeeding interventions globally (Victora et al., 2016). Breastfeeding in the first 2 years could also alleviate one‐third of respiratory infections and half of diarrhoea episodes among children in low‐ and middle‐income countries and may reduce the risks of childhood obesity and non‐communicable diseases later in life (Victora et al., 2016). Beyond the health benefits, there are also economic consequences, with estimated economic losses from suboptimal breastfeeding of about $302 billion annually or 0.49% of world gross national income (Rollins et al., 2016), mainly driven by costs associated with lower cognitive capacity and maternal and child deaths.

Given the importance of breastfeeding, the World Health Organisation (WHO) and UNICEF recommend early initiation of breastfeeding (EIBF) within 1 h of birth, avoidance of prelacteal feeds and exclusive breastfeeding (EBF) for the first 6 months with continued breastfeeding up to 2 years (WHO & UNICEF, 2003). These practices, however, are suboptimal in many parts of the world, with only 42% of infants globally being breastfed within the first hour and 41% exclusively breastfed during their first 6 months of life (UNICEF, 2018).

To address the challenges of suboptimal breastfeeding practices, interventions with various strategies have been implemented and showed positive impacts, particularly in low‐ and middle‐income countries (Haroon, Das, Salam, Imdad, & Bhutta, 2013; Imdad, Yakoob, & Bhutta, 2011). These interventions, however, generally have been implemented at small scale and under relatively controlled conditions, thus their implications for large‐scale programs is uncertain. Alive & Thrive (A&T) is an initiative that aims to improve infant and young child feeding (IYCF) practices through large‐scale social and behaviour change communication interventions, which include interpersonal counselling, mass media and community mobilization, delivered by a non‐governmental organization in Bangladesh and government health systems in Vietnam and Ethiopia (Baker, Sanghvi, Hajeebhoy, Martin, & Lapping, 2013; Piwoz, Baker, & Frongillo, 2013). Over a 4‐year period (2010–2014), the interventions led to large significant impacts on breastfeeding practices in all three countries (Kim et al., 2016; Menon et al., 2016a). Specifically, the impacts were 17 percentage points (pp) for EIBF and 36 pp for EBF in Bangladesh, 10 pp and 28 pp, respectively in Vietnam (Menon et al., 2016a), and 14 and 9 pp respectively in Ethiopia (Kim et al., 2016).

The impact of A&T's interventions on EBF practices may be directly related to intervention exposure during prenatal, intra‐partum and postpartum periods but may also be indirectly related to the influences of interventions on early breastfeeding practices such as EIBF and non‐prelacteal feeding. Interventions to support establishing breastfeeding immediately after birth increase the likelihood of EBF up to 3–6 months of life (WHO, 2017). Mothers experiencing early breastfeeding or positive initial breastfeeds may be more likely to repeat these experiences. A systematic review from low‐ and middle‐income countries showed moderate evidence for EIBF on likelihood of practicing EBF (Kavle, Lacroix, Dau, & Engmann, 2017), with the odds ratio (OR) ranging from 1.4 in Haiti (Walsh, Cordes, Mccreary, & Norr, 2019), 1.7 in Indonesia (Suparmi & Saptarini, 2016), 1.8 in India (Raghavan, Bharti, Kumar, Mukhopadhyay, & Dhaliwal, 2014), to 10.2 in Uganda (Matovu, Kirunda, Rugamba‐Kabagambe, Tumwesigye, & Nuwaha, 2008). Prelacteal feeding delays a newborn's first critical contact with his or her mother, thus disrupting EIBF (UNICEF & WHO, 2018) and is negatively associated with EBF practices (Alzaheb, 2017; Kavle et al., 2017; Sharma & Byrne, 2016). The complex relationships among these three breastfeeding practices (EIBF, prelateal feeds and EBF) and how these relationships are affected by interventions, however, are not known. Having evidence on this can contribute to decision‐making about the importance of focusing on promoting EIBF and non‐prelacteal feeding or on EBF specifically through other strategies. In this paper, we sought to understand the extent to which early breastfeeding practices are associated with EBF and the extent to which intervention‐induced improvements in EIBF and non‐prelacteal feeding contribute to increased prevalence of EBF among infants under 6 months of age.

## 2 METHODS

### 2.1 Study context and data sources

This study is a secondary analysis of data gathered under the overall impact evaluation of A&T's interventions to improve IYCF practices in Bangladesh, Viet Nam and Ethiopia (Menon, Rawat, & Ruel, 2013). The impact evaluations in Bangladesh and Vietnam used a cluster‐randomized design, with two intervention arms: (1) A&T‐intensive areas that received intensified interpersonal counselling, mass media and community mobilization or (2) A&T non‐intensive areas that received standard interpersonal counselling and less‐intensive mass media and community mobilization. In Ethiopia, we used a pre‐ and post‐intervention adequacy evaluation design. We used household survey data that were collected through two cross‐sectional surveys in 2010 and 4 years later (2014) in the same communities (between June and August at both time periods). Overall impact evaluation results have been previously reported (Kim et al., 2016; Menon et al., 2016a; Menon et al., 2016b).

In total, data were collected for ~4000 mothers with children <5 years of age in each round for each country. This paper focuses on EBF; thus, we used household data from mothers with children under 6 months of age (Bangladesh: *n* = 977 at baseline and 998 at endline, Vietnam: *n* = 948 at baseline and 1002 at endline, Ethiopia: *n* = 606 at baseline and 619 at endline). Data were collected via face‐to‐face interviews using structured questionnaires.

### 2.2 Measures

Breastfeeding practices were assessed using the standard WHO indicators (WHO, 2008), on the basis of the maternal recall of all foods and liquids given to children in the first few days after birth and in the 24 h prior to the survey. The outcome was EBF, defined as the proportion of infants 0–5.9 months of age who were fed only breast milk in the previous 24 h (no foods, no liquids with the exception of medications such as drops and syrups; WHO, 2008). Two key explanatory variables were (1) EIBF, defined as the proportion of infants who were reported by mothers to have been put to the breast within 1 h of birth and (2) non‐prelacteal feeding, defined as the proportion of infants who were not fed any foods or liquids other than breastmilk during the first 3 days after birth.

The selection of variables that may influence breastfeeding practices was guided by a conceptual framework of influences on breastfeeding decisions and behaviours (Rollins et al., 2016), considering maternal, child and household levels. Maternal characteristics included age, education, occupation and physical and mental health status. Education represents the highest grade of schooling that mothers completed; different cut‐offs were used for maternal education for the three countries because education levels differ substantially. Occupation is a proxy of workload. In Bangladesh and Ethiopia, we compared housewives with other occupations, whereas in Vietnam, we compared employed mothers with others and examined the influence of returning to work before 6 months after delivery. Physical health was measured by the proportion of mothers with low body mass index (BMI <18.5 kg/m^2^). Mental health was measured by using the Self‐Reporting Questionnaire (WHO, 1994), with each of 20 items scored as 0 or 1 depending on responses related to perceived stresses over the past 30 days. We then created an overall score and used a cut‐point of 7 to classify women as normal or high levels of stress, as suggested by several validation studies (Giang, Allebeck, Kullgren, & Tuan, 2006; Hanlon et al., 2008). Variables at the child level included child age and sex. Household characteristics that may influence breastfeeding practices included number of children <5 years, socio‐economic status (SES) and food security. The SES index was constructed using principal component analysis with variables on ownership of house and land, housing quality and household assets (Gwatkin et al., 2007; Vyas & Kumaranayake, 2006). Household food security was measured using FANTA/USAID's Household Food Insecurity Access Scale (Coates, Swindale, & Bilinsky, 2007).

### 2.3 Statistical analyses

Descriptive analysis was used to examine the characteristics of the study sample. Bivariate analyses were conducted to test the associations between EIBF and EBF and between non‐prelacteal feeding and EBF in each intervention arm at baseline and endline. Multivariable binary logistic regression analyses were used to examine the effects of the interventions and early breastfeeding practices on EBF considering four categories of the intersection between EIBF and the intervention: (1) those who did not practice EIBF in non‐intensive areas, (2) those who practiced EIBF in non‐intensive areas, (3) those who did not practice EIBF in intensive areas and (4) those who practiced EIBF in intensive areas. Similar categories were made for the combinations of non‐prelacteal feeding and intervention area. All models controlled for variables at the maternal, child and household levels as described above and accounted for clustering at village, commune or district levels using a robust sandwich estimator of the standard errors. Structural equation modelling was used to assess the relationship between interventions with breastfeeding practices and to quantify how much of the intervention impact on EBF was direct and how much was indirect via improving EIBF and non‐prelacteal feeding. The models assumed that the intervention could affect EIBF, no‐prelacteal feeding and EBF; EIBF could affect no‐prelacteal feeding and EBF, and no‐prelacteal feeding could affect EBF. No important interactions were found among intervention, EIBF and non‐prelacteal feeding. All analysis was done using Stata version 15.1 software (StataCorp, 2009). Statistical significance was defined as *p* value < 0.05.

## 3 RESULTS

### 3.1 Sample characteristics

The study samples from three countries shared similarities in maternal age and child age and sex (Table 1). Maternal education level was highest for Vietnam (all women had some education, and more than one‐third completed high school level or higher), followed by Bangladesh (23% of women had no education) and lowest for Ethiopia (60% of women had no education). The percentages of housewives were 95% for Bangladesh, 53% for Ethiopia and 13% for Vietnam. Maternal stress was highest in Bangladesh (43%), followed by Ethiopia (36%) and Vietnam (29%). Household food insecurity was highly prevalent in Ethiopia (62%).

**TABLE 1:** Selected characteristics of the study samples in Bangladesh, Vietnam and Ethiopia at baseline

Characteristic	Bangladesh		Vietnam		Ethiopia
	Intensive (*n =* 487)	Non‐intensive (*n =* 490)	Intensive (*n =* 488)	Non‐intensive (*n =* 460)	Total (*n =* 606)
**Maternal characteristics**					
Mean age (years)	25.59 ± 6.21	24.54 ± 5.49	27.16 ± 5.46	27.14 ± 5.28	27.87 ± 6.17
Occupation (%)					
Housewife	96.10	94.69	14.96	11.09	53.47
Farmer	–	–	51.64	51.52	39.60
Salary employee/Self‐employed	3.90	5.32	33.40	37.39	6.93
Education (%)					
No schooling	24.44	21.43	2.41	1.23	60.30
Primary school	29.57	27.14	10.50	12.35	26.25
Secondary school	39.63	42.45	52.52	49.18	9.30
High school or higher	6.37	8.98	34.57	37.24	3.32
Underweight^a^ (%)	24.90	26.12	20.08	22.44	16.72
Mental stress score ≥ 7 (%)	43.53	41.63	26.64	31.52	35.64
**Child characteristics**					
Mean age (months)	3.39 ± 1.61	3.30 ± 1.60	3.55 ± 1.48	3.53 ± 1.49	2.92 ± 1.62
Female (%)	50.10	50.20	45.70	48.91	50.91
**Household characteristics**					
Household food security^b^ (%)	68.79	69.80	70.08	65.14	37.95
Ownership of house (%)	94.87	93.88	39.34	38.91	93.07
Ownership of agricultural land (%)	49.28	39.80^*^	71.72	73.04	88.28
Ownership of garden (%)	27.52	33.06	66.05	61.66	52.48
Mean socioeconomic index^c^	0.05 ± 1.05	0.15 ± 1.15	−0.27 ± 0.97	−0.27 ± 0.85	−0.14 ± 0.84

*Note.* Values are mean ± standard deviation (SD) or per cent. *p* values obtained from models adjusted for clustering effect at commune and district levels.

a Body mass index < 18.5 kg/m^2^.

b Household food security was measured using FANTA/USAID's Household Food Insecurity Access Scale.

c Socio‐economic status (SES) index was constructed using principal components analysis with variables on ownerships and assets. It is standardized score with mean = 0 and SD = 1.

* *p* < 0.05.

### 3.2 Breastfeeding practices

**FIGURE 1:**
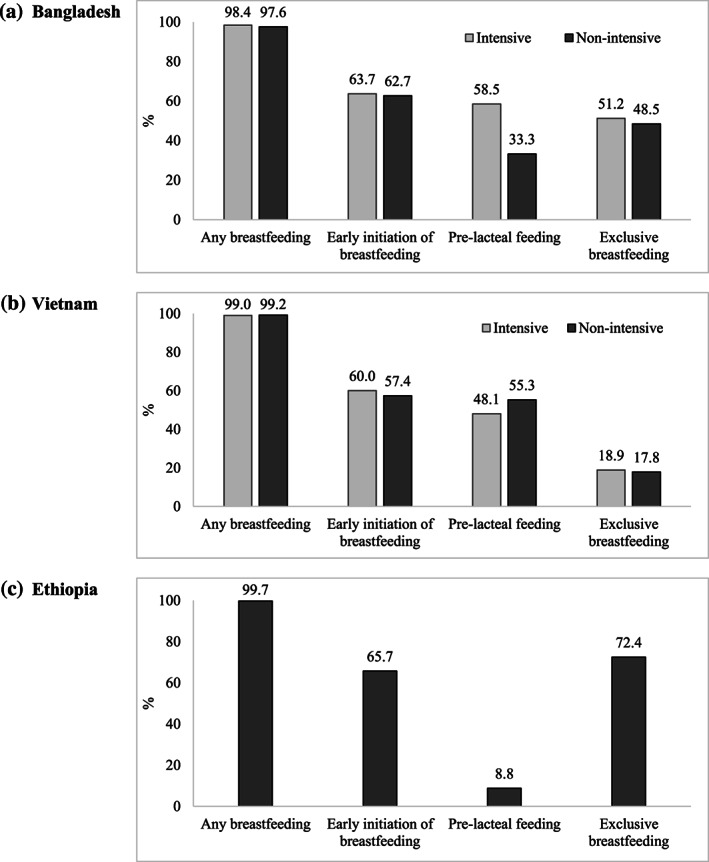
Breastfeeding practices in (a) Bangladesh, (b) Vietnam and (c) Ethiopia at baseline

Although breastfeeding is nearly universal in all three countries (≥98%), delayed initiation of breastfeeding is still prevalent with only about two‐thirds of infants being put to the breast within an hour of birth (Figure 1). Prelacteal feeding was common, practiced by nearly a half of the respondents in Bangladesh and Vietnam. EBF was highest in Ethiopia (72%) and lowest in Vietnam (18%).

### 3.3 Association between EIBF and EBF

Results from multivariable logistics regression models showed that at baseline, EIBF was not associated with higher odds of EBF, compared with those who did not practice EIBF, in any of the countries (Table 2). At endline, EIBF alone was not associated with EBF, but women living in the intensive intervention areas were 3.5–3.9 times more likely to practice EBF in Bangladesh and Vietnam. The association between EIBF and EBF was particularly large among those living in intensive areas (OR = 11 for Bangladesh and OR = 6.5 for Vietnam), compared with those with no EIBF and living in non‐intensive areas.

**TABLE 2:** Regression models for the association between early initiation of breastfeeding and exclusive breastfeeding among children <6 months in Bangladesh, Vietnam and Ethiopia

Model	Bangladesh		Vietnam		Ethiopia	
	Baseline	Endline	Baseline	Endline	Baseline	Endline
No EIBF, non‐intensive	Ref.	Ref.	Ref.	Ref.	Ref.	Ref.
EIBF, non‐intensive	1.12 (0.79, 1.58)	0.89 (0.51, 1.57)	1.03 (0.71, 1.48)	1.22 (0.80, 1.87)	1.11 (0.73, 1.70)	–
No EIBF, intensive	–	3.91^*^ (1.04, 14.69)	–	3.54^***^ (2.21, 5.66)	–	–
Both EIBF and intensive	–	10.95^***^ (5.40, 22.19)	–	6.47^***^ (3.85, 10.86)	–	1.41 (0.75, 2.63)

*Note.* Values are odd ratios [95% confidence interval (CI)]. All models adjusted for child age, sex, maternal age, education, occupation, body mass index (BMI), mental stress, household food security, socio‐economic status (SES) and clustering effect at village or commune and district levels.

Abbreviation: EIBF, early initiation of breastfeeding.

* *p* < 0.05.

*** *p* < 0.001.

### 3.4 Association between prelacteal feeding and EBF

At baseline, those who did not practice‐prelacteal feeding had 1.6–3.5 higher odds of EBF, compared with those with prelacteal feeding, across the three countries (Table 3). At endline, those with non‐prelacteal feeding and living in non‐intensive areas or prelacteal feeding and in intensive areas were 2–3 times more likely to practice EBF, compared with those who practiced prelacteal feeding and lived in non‐intensive areas in Bangladesh and Vietnam. The odds of EBF were particularly high among mothers who did not practice prelacteal feeding and lived in intensive areas (OR = 27.5 in Bangladesh and 11.5 in Vietnam).

**TABLE 3:** Regression models for the association between non‐prelacteal feeding and exclusive breastfeeding among children <6 months in Bangladesh, Vietnam and Ethiopia

Model	Bangladesh		Vietnam		Ethiopia	
	Baseline	Endline	Baseline	Endline	Baseline	Endline
Prelacteal feeding, non‐intensive	Ref.	Ref.	Ref.	Ref.	Ref.	Ref.
Non‐prelacteal feeding, non‐intensive	1.55^**^ (1.14, 2.12)	2.09^***^ (1.44, 3.05)	3.46^***^ (2.23, 5.38)	2.56^***^ (1.77, 3.69)	2.01^+^ (0.99, 4.05)	–
Prelacteal feeding, intensive	–	2.14^*^ (1.03, 4.45)	–	3.02^***^ (1.77, 3.69)	–	–
Both non‐prelacteal feeding and intensive	–	27.50^***^ (15.74, 48.04)	–	11.52^***^ (7.12, 18.66)	–	2.88+ (0.95, 8.68)

*Note.* Values are odd ratios [95% confidence interval (CI)]. All models adjusted for child age, sex, maternal age, education, occupation, body mass index (BMI), mental stress, household food security, socio‐economic status (SES) and clustering effect at village or commune and district level.

+ *p* < 0.10.

* *p* < 0.05.

** *p* < 0.01.

*** *p* < 0.001.

### 3.5 Direct and indirect effects from intervention exposure to EBF

**FIGURE 2:**
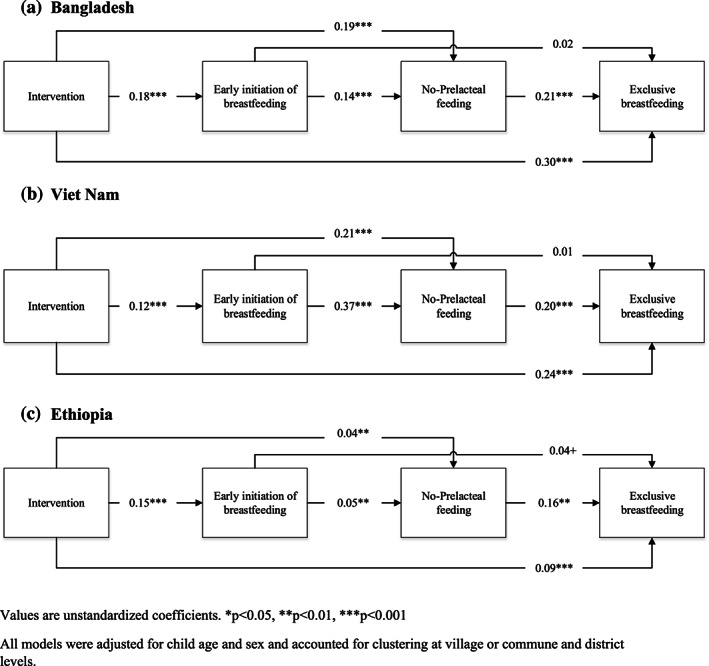
Path models for the associations from intervention group and early breastfeeding practices to exclusive breastfeeding at endline, (a) Bangladesh, (b) Vietnam and (c) Ethiopia

Living in intervention area had significant direct effects on each of the breastfeeding practices in all three countries (Figure 2). For EIBF, the intervention effects were highest for Bangladesh (β = 0.18; *p* < 0.001), followed by Ethiopia (β = 0.15; *p* < 0.001) and Vietnam (β = 0.12; *p* < 0.001). For non‐prelacteal feeding, the intervention had similarly large effects in Bangladesh and Vietnam (β = 0.19–0.21; *p* < 0.001) and a smaller effect in Ethiopia (β = 0.04; *p* < 0.01). In all three countries, EIBF was positively associated with non‐prelacteal feeding (β = 0.05–0.37; all *p* < 0.01), and non‐prelacteal feeding, in turn, was associated with higher EBF (β = 0.16–0.17; all *p* < 0.01). For Bangladesh, the total effect from intervention exposure to EBF was 0.344, with 13% being indirect effect through EIBF and non‐prelacteal feeding. For Vietnam, the total effect from intervention to EBF was 0.292, with 18% being indirect through EIBF and non‐prelacteal feeding. For Ethiopia, the total effect from intervention to EBF was 0.105, with 14% being indirect through EIBF and non‐prelacteal feeding. Across the three countries, the paths through EIBF and non‐prelacteal feeding explained 13%–18% of the effect of the intervention on EBF; the models fit well, with the root mean square errors of approximation and the standardized root mean square residuals all less than 0.001, the comparative fit indices all above 0.999, and model chi‐square statistics all greater than 95.

## 4 DISCUSSION

Using data from three impact evaluations of large‐scale social and behaviour change communication interventions, we observed that EIBF alone was not associated with EBF, whereas non‐prelacteal feeding was associated with 1.6–3.5 higher odds of EBF. There was positive intervention impact across the three countries on breastfeeding practices, with large amplified effects on EBF among those who practiced EIBF or avoided prelacteal feeding. Thus, the path of influence from EIBF to non‐prelacteal feeding to EBF was important, explaining 13%–18% of the intervention effect on EBF.

Although EIBF alone was not associated with EBF, EIBF immediately after birth may be important for establishing any breastfeeding practice and assuring that breastmilk is the first food introduced to infants. Given that nearly all mothers reported any breastfeeding in the three countries, early to later breastfeeding practices are intertwined more tightly than our analyses may show. On the other hand, the relationship between non‐prelacteal feeding in the first 3 days after birth and EBF was reflected in our study results. Establishing exclusivity (or feeding nothing else aside from breastmilk) in the first 3 days appeared important for establishing EBF; this early practice likely set the foundation or formed a habit for maintaining the practice in the first 6 months of the child's life (UNICEF & WHO, 2018). Inasmuch as EIBF was associated with non‐prelacteal feeding and non‐prelacteal feeding was strongly linked to EBF, both early breastfeeding practices were influential for EBF.

Living in intervention areas was above all the most influential on breastfeeding practices, particularly EBF. Our results showed positive effects of intervention exposure directly on each breastfeeding practice, thus social and behaviour change interventions should be targeted at EIBF, non‐prelacteal feeding, and EBF to promote each of these practices. Interventions aimed only at promoting early breastfeeding practices, however, should not be expected to have a large effect on EBF. A recent review on programmes and interventions to support optimal breastfeeding in South Asia reported that Maternal, Newborn Child Health initiatives were effective at supporting EIBF and avoiding prelacteal feeding but had mixed results for EBF (Benedict, Craig, Torlesse, & Stoltzfus, 2018). In rural Bangladesh, providing traditional birth attendants and community volunteers with several days of training on the WHO breastfeeding curriculum led to increased EIBF and avoidance of prelacteal feeds, but did not have impact on EBF (Talukder, Farhana, Vitta, & Greiner, 2017). The Kangaroo Mother Care intervention for very low birth weight infants in India reported positive EIBF findings, yet there was no impact on EBF at 6 months (Gavhane, Eklare, & Mohammad, 2016). Conditional cash transfers to increase demand for facility‐based delivery and reproductive health services, including counselling on breastfeeding in India also had positive effects on EIBF but not on EBF (Carvalho, Thacker, Gupta, & Salomon, 2014). These interventions offered breastfeeding support immediately following delivery and during the postnatal period and thus had the most opportunity to impact on early feeding behaviours. EBF is a practice that lasts over a 6‐month period and requires continual support, so interventions aimed only at the initiation and early days of breastfeeding will be inadequate to sustain EBF practice. Still, in our study, the magnitude of effect on EBF was greatest for those women living in intervention areas and also had appropriate early breastfeeding practice.

Social and behaviour change interventions played an important role along the path from early breastfeeding practices to EBF, but the context of where these interventions are implemented must also be considered. In a separate paper, we examined the contextual characteristics, features of the promoted behaviours, reach of the delivery platform and the potential to benefit and observed that these together contributed to the adoption of the promoted child feeding practices (Menon et al., 2017). For instance, the effects of early breastfeeding practices and even intervention exposure on EBF were attenuated in Ethiopia, compared with Bangladesh and Vietnam, where EBIF and EBF prevalence was highest and prelacteal feeding was low; thus, the potential to benefit on breastfeeding practices was lowest in Ethiopia. Another feature of intervention exposure that affects impact is the combination and intensity of interventions, and those required to achieve behaviour change may be context‐specific (Kim et al., 2020).

We used data from large‐scale intervention evaluations in three countries to examine the influence of the intervention on the complex relationships among EIBF, avoidance of prelacteal feeding and EBF. Breastfeeding practices were measured by standard study methods (WHO, 2008) and similar questionnaires, which allowed comparing across countries. The structural equation models quantified the extent to which EIBF and non‐prelacteal feeding contribute to EBF in different country contexts. In using cross‐sectional surveys among mothers with children less than 6 months of age to assess breastfeeding practices at two time points, we did not follow breastfeeding practices among the same women over time. Although we have data on past and current breastfeeding practices among the same women, we cannot determine the progression of how early breastfeeding practices influenced EBF in the same women or how intervention exposure during earlier practices may or may not have led to current practices. Given the long duration of intervention implementation (over 3 years) in the same communities, however, study mothers living in the intervention areas would have been exposed throughout pregnancy and after birth, giving us a cohort of women to examine the different breastfeeding practices at the same time. Breastfeeding practices were based on mothers' report, which may be subject to recall bias. Given that all mothers reported on feeding practices that were ongoing or within the last 6 months, we believe mothers would have had good recall.

Understanding the extent to which EIBF and non‐prelacteal feeding contribute to EBF in different country contexts provides important insights on how to prioritize intervention strategies. Our study results showed that early breastfeeding practices matter in influencing EBF, but interventions aimed only at the initiation and early days of breastfeeding will be inadequate to sustain EBF practice. Social and behaviour change interventions should be simultaneously targeted at EIBF, non‐prelacteal feeding and EBF to support full range of optimal breastfeeding practices.

## CONFLICTS OF INTEREST

The authors declare that hey have no conflicts of interest.

## CONTRIBUTIONS

PHN, SSK, PM and EAF designed the overall study. PHN and SSK developed the draft of the manuscript. PHN and LMT conducted the data analysis. EAF and PM critically reviewed and revised the manuscript. PHN made the final revisions. All authors read and approved the final version of the paper.

## References

[mcn13012-bib-0001] Alzaheb, R. A. (2017). A review of the factors associated with the timely initiation of breastfeeding and exclusive breastfeeding in the Middle East. Clin Med Insights Pediatr, 11, 1179556517748912. 29317851 10.1177/1179556517748912 PMC5753894

[mcn13012-bib-0002] Baker, J. , Sanghvi, T. , Hajeebhoy, N. , Martin, L. , & Lapping, K. (2013). Using an evidence‐based approach to design large‐scale programs to improve infant and young child feeding. Food and Nutrition Bulletin, 34, S146–S155. 10.1177/15648265130343S202 24261073

[mcn13012-bib-0003] Benedict, R. K. , Craig, H. C. , Torlesse, H. , & Stoltzfus, R. J. (2018). Effectiveness of programmes and interventions to support optimal breastfeeding among children 0‐23 months, South Asia: A scoping review. Matern Child Nutr, 14(Suppl 4), e12697. 30499251 10.1111/mcn.12697 PMC6519148

[mcn13012-bib-0004] Carroll, G. J. , Buccini, G. S. , & Perez‐Escamilla, R. (2018). Perspective: What will it cost to scale‐up breastfeeding programs? A comparison of current global costing methodologies. Advances in Nutrition, 9, 572–580. 10.1093/advances/nmy041 30060074 PMC6140429

[mcn13012-bib-0005] Carvalho, N. , Thacker, N. , Gupta, S. S. , & Salomon, J. A. (2014). More evidence on the impact of India's conditional cash transfer program, Janani Suraksha Yojana: Quasi‐experimental evaluation of the effects on childhood immunization and other reproductive and child health outcomes. PLoS ONE, 9, e109311. 25303078 10.1371/journal.pone.0109311 PMC4193776

[mcn13012-bib-0006] Coates, J. , Swindale, A. , & Bilinsky, P. (2007). Household Food Insecurity Access Scale (HFIAS) for measurement of household food access: Indicator guide (v. 3). Washington, D.C.: Food and Nutrition Technical Assistance Project, Academy for Educational Development.

[mcn13012-bib-0007] Gavhane, S. , Eklare, D. , & Mohammad, H. (2016). Long term outcomes of kangaroo mother care in very low birth weight infants. Journal of Clinical and Diagnostic Research, 10, SC13–SC15. 10.7860/JCDR/2016/23855.9006 PMC5296538 28208965

[mcn13012-bib-0008] Giang, K. B. , Allebeck, P. , Kullgren, G. , & Tuan, N. V. (2006). The Vietnamese version of the Self Reporting Questionnaire 20 (SRQ‐20) in detecting mental disorders in rural Vietnam: A validation study. The International Journal of Social Psychiatry, 52, 175–184. 10.1177/0020764006061251 16615249

[mcn13012-bib-0009] Gwatkin, D. , Rutstein, S. , Johnson, K. , Suliman, E. , Wagstaff, A. , & Amouzou, A. (2007). Socio‐economic differences in health, nutrition, and population within developing countries: An overview. Nigerian Journal of Clinical Practice, 10, 272–282. 18293634

[mcn13012-bib-0010] Hanlon, C. , Medhin, G. , Alem, A. , Araya, M. , Abdulahi, A. , Hughes, M. , … Prince, M. (2008). Detecting perinatal common mental disorders in Ethiopia: Validation of the self‐reporting questionnaire and Edinburgh Postnatal Depression Scale. Journal of Affective Disorders, 108, 251–262. 10.1016/j.jad.2007.10.023 18055019

[mcn13012-bib-0011] Haroon, S. , Das, J. K. , Salam, R. A. , Imdad, A. , & Bhutta, Z. A. (2013). Breastfeeding promotion interventions and breastfeeding practices: A systematic review. BMC Public Health, 13(Suppl 3), S20. 24564836 10.1186/1471-2458-13-S3-S20 PMC3847366

[mcn13012-bib-0012] Imdad, A. , Yakoob, M. Y. , & Bhutta, Z. A. (2011). Effect of breastfeeding promotion interventions on breastfeeding rates, with special focus on developing countries. BMC Public Health, 11(Suppl 3), S24. 21501442 10.1186/1471-2458-11-S3-S24 PMC3231898

[mcn13012-bib-0013] Kavle, J. A. , Lacroix, E. , Dau, H. , & Engmann, C. (2017). Addressing barriers to exclusive breast‐feeding in low‐ and middle‐income countries: A systematic review and programmatic implications. Public Health Nutrition, 20, 3120–3134. 10.1017/S1368980017002531 28965508 PMC10262277

[mcn13012-bib-0014] Kim, S. S. , Nguyen, P. H. , Tran, L. M. , Alayon, S. , Menon, P. , & Frongillo, E. A. (2020). Combination of behavior change interventions and frequency of interpersonal communication associated with infant and young child feeding practices are context‐specific in Bangladesh, Ethiopia, and Viet Nam. Current Development of Nutrition, 4, nzz140. 10.1093/cdn/nzz140 PMC6964730 31976385

[mcn13012-bib-0015] Kim, S. S. , Rawat, R. , Mwangi, E. M. , Tesfaye, R. , Abebe, Y. , Baker, J. , … Menon, P. (2016). Exposure to large‐scale social and behavior change communication interventions is associated with improvements in infant and young child feeding practices in Ethiopia. PLoS ONE, 11, e0164800 10.1371/journal.pone.0164800 27755586 PMC5068829

[mcn13012-bib-0016] Matovu, A. , Kirunda, B. , Rugamba‐Kabagambe, G. , Tumwesigye, N. M. , & Nuwaha, F. (2008). Factors influencing adherence to exclusive breast feeding among HIV positive mothers in Kabarole District, Uganda. East African Medical Journal, 85, 162–170. 10.4314/eamj.v85i4.9640 18700349

[mcn13012-bib-0017] Menon, P. , Nguyen, P. H. , Kim, S. , Tran, L. M. , Frongillo, E. A. , Ruel, M. , Rawat, R. (2017). Context Matters: Insights from Two Randomized Evaluations of Behavior Change Interventions on Factors Influencing Infant and Young Child Feeding Practices in Bangladesh and Vietnam The Faseb Journal, 3(1), Supplement. https://www.fasebj.org/doi/abs/10.1096/fasebj.31.1_supplement.165.6.

[mcn13012-bib-0018] Menon, P. , Nguyen, P. H. , Saha, K. K. , Khaled, A. , Kennedy, A. , Tran, L. M. , … Rawat, R. (2016a). Impacts on breastfeeding practices of at‐scale strategies that combine intensive interpersonal counseling, mass media, and community mobilization: Results of cluster‐randomized program evaluations in Bangladesh and Viet Nam. PLoS Medicine, 13, e1002159 10.1371/journal.pmed.1002159 27780198 PMC5079648

[mcn13012-bib-0019] Menon, P. , Nguyen, P. H. , Saha, K. K. , Khaled, A. , Sanghvi, T. , Baker, J. , … Rawat, R. (2016b). Combining intensive counseling by frontline workers with a nationwide mass media campaign has large differential impacts on complementary feeding practices but not on child growth: Results of a cluster‐randomized program evaluation in Bangladesh. The Journal of Nutrition, 146, 2075–2084. 10.3945/jn.116.232314 27581575 PMC5037872

[mcn13012-bib-0020] Menon, P. , Rawat, R. , & Ruel, M. (2013). Bringing rigor to evaluations of large‐scale programs to improve infant and young child feeding and nutrition: The evaluation designs for the Alive & Thrive initiative. Food and Nutrition Bulletin, 34, S195–S211. 10.1177/15648265130343S206 24261077

[mcn13012-bib-0021] Piwoz, E. , Baker, J. , & Frongillo, E. A. (2013). Documenting large‐scale programs to improve infant and young child feeding is key to facilitating progress in child nutrition. Food and Nutrition Bulletin, 34, S143–S145. 10.1177/15648265130343S201 24261072

[mcn13012-bib-0022] Raghavan, V. , Bharti, B. , Kumar, P. , Mukhopadhyay, K. , & Dhaliwal, L. (2014). First hour initiation of breastfeeding and exclusive breastfeeding at six weeks: Prevalence and predictors in a tertiary care setting. Indian Journal of Pediatrics, 81, 743–750. 10.1007/s12098-013-1200-y 24113879

[mcn13012-bib-0023] Rollins, N. C. , Bhandari, N. , Hajeebhoy, N. , Horton, S. , Lutter, C. K. , Martines, J. C. , … Lancet Breastfeeding Series Group . (2016). Why invest, and what it will take to improve breastfeeding practices? Lancet, 387, 491–504. 10.1016/S0140-6736(15)01044-2 26869576

[mcn13012-bib-0024] Sharma, I. K. , & Byrne, A. (2016). Early initiation of breastfeeding: A systematic literature review of factors and barriers in South Asia. International Breastfeeding Journal, 11, 17. 27330542 10.1186/s13006-016-0076-7 PMC4912741

[mcn13012-bib-0025] Statacorp . (2009). Stata Statistical Software: Release 11. College Station. Texas 77845 USA: Copyright 2009 StataCorp LP.

[mcn13012-bib-0026] Suparmi, S. , & Saptarini, I. (2016). Early initiation of breast feeding but not bottle feeding increase exclusive breastfeeding practice among less than six months infant in Indonesia. Health Science Journal of Indonesia, 7(1), 44–48.

[mcn13012-bib-0027] Talukder, S. , Farhana, D. , Vitta, B. , & Greiner, T. (2017). In a rural area of Bangladesh, traditional birth attendant training improved early infant feeding practices: A pragmatic cluster randomized trial. Matern Child Nutr, 13 10.1111/mcn.12237 PMC6865915 26775711

[mcn13012-bib-0028] UNICEF . (2018). UNICEF Global Databases. Washington DC, USA: Nutrition: Infant and Young Child Feeding https://data.unicef.org/topic/nutrition/infant-and-young-child-feeding/

[mcn13012-bib-0029] UNICEF & WHO . (2018). Capture the moment—Early initiation of breastfeeding: The best start for every newborn. New York: UNICEF.

[mcn13012-bib-0030] Victora, C. G. , Bahl, R. , Barros, A. J. D. , França, G. V. A. , Horton, S. , Krasevec, J. , … Rollins, N. C. (2016). Breastfeeding in the 21st century: Epidemiology, mechanisms, and lifelong effect. Lancet, 387(10017), 475–490. 10.1016/S0140-6736(15)01024-7 26869575

[mcn13012-bib-0031] Vyas, S. , & Kumaranayake, L. (2006). Constructing socio‐economic status indices: How to use principal components analysis. Health Policy and Planning, 21, 459–468. 10.1093/heapol/czl029 17030551

[mcn13012-bib-0032] Walsh, S. M. , Cordes, L. , Mccreary, L. , & Norr, K. F. (2019). Effects of early initiation of breastfeeding on exclusive breastfeeding practices of mothers in Rural Haiti. Journal of Pediatric Health Care, 33, 561–567. 10.1016/j.pedhc.2019.02.010 31153727

[mcn13012-bib-0033] WHO . (1994). A user's guide to self‐reporting questionnaires. Geneva: Division of mental health, World Health Organization.

[mcn13012-bib-0034] WHO . (2008). Indicators for assessing infant and young child feeding practices. Part I: Definition Geneva: WHO Press.

[mcn13012-bib-0035] WHO . (2017). Guideline: Protecting, promoting and supporting breastfeeding in facilities providing maternity and newborn services. Geneva: World Health Organization. Licence: CC BY‐NC‐SA 3.0 IGO. 29565522

[mcn13012-bib-0036] WHO & UNICEF . (2003). Global strategy for infant and young child feeding. https://www.who.int/nutrition/publications/infantfeeding/9241562218/en/ Geneva: Switzerland, World Health Organization.

